# Neoplastic Meningitis: How MRI and CSF Cytology Are Influenced by CSF Cell Count and Tumor Type

**DOI:** 10.1155/2013/248072

**Published:** 2013-12-19

**Authors:** P. Prömmel, S. Pilgram-Pastor, H. Sitter, J.-H. Buhk, H. Strik

**Affiliations:** ^1^Department of Neurology, Medical School, University of Goettingen, Germany; ^2^Department of Neurosurgery, University of Zurich, Switzerland; ^3^Department of Neuroradiology, Medical School, University of Goettingen, Germany; ^4^Institute of Surgical Research, University of Marburg, Baldinger Stra**β**e, 35043 Marburg, Germany; ^5^Department of Neuroradiology, University of Hamburg, Germany; ^6^Department of Neurology, University of Marburg, Baldinger Stra**β**e, 35043 Marburg, Germany

## Abstract

*Background*. Although CSF cytology and MRI are standard methods to diagnose neoplastic meningitis (NM), this complication of neoplastic disease remains difficult to detect. We therefore reevaluated the sensitivity of gadolinium (GD)-enhanced MRI and cerebrospinal-fluid (CSF)-cytology and the relevance of tumor type and CSF cell count. *Methods*. We retrospectively identified 111 cases of NM diagnosed in our CSF laboratory since 1990 with complete documentation of both MRI and CSF cytology. 37 had haematological and 74 solid neoplasms. CSF cell counts were increased in 74 and normal in 37 patients. *Results*. In hematological neoplasms, MRI was positive in 49% and CSF cytology in 97%. In solid tumors, the sensitivity of MRI was 80% and of cytology 78%. With normal CSF cell counts, MRI was positive in 59% (50% hematological, 72% solid malignancies) and CSF cytology in 76% (92% in hematological, 68% in solid neoplasms). In cases of elevated cell counts, the sensitivity of MRI was 72% (50% for hematological, 83% for solid malignancies) and of CSF cytology 91% (100% for haematological and 85% for solid neoplasms). 91% of cytologically positive cases were diagnosed at first and another 7% at second lumbar puncture. Routine protein analyses had a low sensitivity in detecting NM. *Conclusions*. The high overall sensitivity of MRI was only confirmed for NM from solid tumors and for elevated CSF cell counts. With normal cell counts and haematological neoplasms, CSF-cytology was superior to MRI. None of the analysed routine CSF proteins had an acceptable sensitivity and specificity in detecting leptomeningeal disease.

## 1. Introduction

Neoplastic meningitis (NM) is a diffuse spreading of malignant cells into the cerebrospinal fluid (CSF) and/or into the adjacent leptomeninges. The CSF flow can distribute the malignant cells throughout the entire central nervous system (CNS) causing a variety of clinical symptoms. Early diagnosis of NM is regarded to be crucial since the rapid institution of therapy may offer the best chances for successful treatment [1–3].

In autopsy series of patients with malignant disease, leptomeningeal involvement was diagnosed in up to 19% [4]. Even in these days of modern sectional imaging and cytological diagnostics, NM is diagnosed in only 5–15% of living patients with malignant disease [1, 5]. This discrepancy in the frequency of NM indicates that NM is underdiagnosed and that there is a need for reevaluation of the standard diagnostic procedures.

In solid tumors, NM occurs in about 5–8% of patients, most commonly with carcinomas of the breast and lung and melanoma [1–3]. Among the haematological neoplasms, aggressive lymphomas of B-cell origin affect the meninges in 5–15% [6] and acute lymphatic leukemias (ALL) in 1–10% of cases. Primary CNS neoplasms of astrocytic and oligodendroglial origin only rarely involve the meninges, with the exception of the infrequently occurring spinal localizations [7]. In the rare ependymomas, medulloblastomas and primary CNS lymphomas, however, CSF spread is often observed [8].

Neoplastic meningitis may occur diffusely with malignant cells floating freely within the CSF or as adherent types [9]. While CSF cytology is expected to detect diffuse CSF involvement, MRI should visualize meningeal contrast enhancement caused by adherent types. Although both forms of leptomeningeal involvement often occur combined, either type may prevail with impact on the appropriate diagnostic method. In this retrospective series, we compared the diagnostic sensitivity of both methods when performed with high quality and the additional value of standard CSF analysis.

## 2. Methods

### 2.1. Patients and Diagnostic Procedures

The archives of the Laboratory of Neurochemistry, the Department of Neuroradiology, and the general diagnostic database of the University Medicine Göttingen were searched for patients with neoplastic meningitis of which both CSF cytology and MRI of the CNS were available. Only cases with complete documentation of the radiological and cytological examinations were included. Between 1994 and 2009, 111 evaluable cases of NM were identified.

The diagnosis of NM was proved by review of the cytospin slides (HS, PP, and IN) and MR-images (JHB, SPP, and PP) and review of the clinical files with regard to clinical symptoms and further clinical course. T1-weighted images with and without gadolinium and FLAIR-sequences were used for the reevaluation of the MR-imaging. Radiological criteria of NM were signal alterations of the meninges and contrast enhancement lining the CSF spaces. Only irregular or nodular contrast enhancement of the meninges was considered suspicious of NM in order to avoid misdiagnosing fine linear meningeal enhancement which is known to occur as an unspecific finding after lumbar puncture [10–12]. Cytological signs of malignant CSF disease were the standard criteria such as abnormally increased size, irregular forms of cell or nucleus, or staining patterns [13]. The review of the exams served to exclude false positive cases. No additional cases were included retrospectively which had not been detected initially. In addition, all other routine parameters of CSF examination were documented: total cell count, total protein, intrathecal immunoglobulin synthesis, oligoclonal bands, lactate, and ferritin. In 45 cases, MRI was performed before and in 48 cases after lumbar puncture. In 18 patients, both procedures were done on the same day, and the exact sequence of the examinations could not be assessed retrospectively.

### 2.2. Statistical Analysis

For each case, the diagnostic method leading to the correct diagnosis (cytology, MRI, or both) was assessed. In addition, the number of CSF probes necessary to establish the cytological diagnosis was analysed. The diagnostic sensitivity was calculated as percentage of positive diagnoses of the total number of cases. This was calculated separately for each diagnostic method in the whole series and for subgroups with normal and elevated cell count and with haematological or solid neoplasms.

## 3. Results 

Between 1994 and 2009, 111 cases of NM were identified: 37 patients had hematological and 74 solid neoplasms. CSF cell counts were elevated in 74 and normal in 37 patients.

In the whole series, cytology was significantly more sensitive than MRI (Table 1). In particular in hematological neoplasms, CSF cytology detected NM significantly more often. In solid tumors, by contrast, the sensitivities were not different.

CSF cell counts influenced the overall diagnostic sensitivity considerably: CSF-cytology detected neoplastic meningitis more often in cases with CSF pleocytosis than with normal cell counts. Equally, MRI was more often positive with elevated than with normal cell counts. In haematological neoplasms with higher cell counts, CSF cytology remained to be significantly more sensitive than MRI. Of note, we could not detect MRI-positive, cytology negative haematological NM with elevated cell counts in our databases. Thus, we consider the sensitivity of cytology in this specific subgroup to be artificially high.

In the whole series with positive CSF cytology, 91% were already detected with the first lumbar puncture. The second CSF sample revealed additional 7% of cases and the third and all additional punctures added only 2% more positive cases. In 11 patients, the first CSF probe was cytologically negative, and no further CSF samples were taken because MRI and clinical symptoms clearly indicated NM. This translates into an overall sensitivity of 79% of all cases with the first puncture, 85% with the second and 86% with three or more samples.

Alterations of CSF proteins were variable (Table 2): total protein, lactate, and ferritin were elevated in only 40–70% and IgG was oligoclonal in 42%. Taken together, at least one of these parameters was pathological in 84% of cases.

## 4. Discussion

Although a well-known complication of neoplastic disease, the early diagnosis of neoplastic meningitis is often difficult even in these times of modern diagnostic methods. This retrospective analysis found comparable sensitivities of CSF cytology and MRI for solid neoplasms, but a superiority of CSF cytology in hematological malignancies. CSF pleocytosis largely influenced the diagnostic accuracy. The analysis of standard CSF proteins has no positive diagnostic value, but negativity of all standard parameters (cell count, total protein, lactate, ferritine, and oligoclonal bands) is associated with a probability of over 80% that NM is excluded.

While the occurrence of CNS relapse in hematological malignancies has dropped considerably after the introduction of CNS prophylaxis, [14, 15] the frequency of leptomeningeal involvement by systemic *solid *tumors has increased in recent years [16]. This is attributable in particular to the better control of the systemic disease with chemotherapeutic agents or modern targeted substances, many of which are unable to cross the blood-brain barrier [17]. As a result, recurrence more often affects the CNS, as observed in the striking example of Her2-positive breast cancer following antibody therapy with trastuzumab. In this entity, about 30% of recurrences manifest as CNS metastases and 20% as NM [18].

Since early diagnosis and onset of treatment are regarded to be crucial for successful treatment and prevention of permanent neurological sequelae, [19] the early detection of NM is of major importance. The introduction of MRI in the diagnosis of CNS disease with high-resolution imaging of the leptomeninges has enhanced the sensitivity for the detection of NM enormously as compared with computed tomography. With reported sensitivities of more than 70%, [20, 21] MRI is presumed to reach a similar or even better diagnostic accuracy than conventional CSF cytology for which sensitivities of 54 to 90% have been reported [3, 4]. In addition to NM, at least one-third of patients have solid CNS metastases [3, 22]. Thus, MRI of the whole CNS—brain and spine—is mandatory in cases of suspected or proven NM. It might therefore be questioned whether CSF cytology is still necessary in view of such a good diagnostic accuracy of MRI.

The analysis presented here proves that in fact MRI is highly sensitive in detecting NM from solid neoplasms, especially in cases of elevated cell counts. In line with other studies, [23, 24] we observed the best detection of leptomeningeal involvement with contrast-enhanced T1-weighted MRI. Approximately 20% of NM from solid tumors, however, were MRI-negative and only detected by CSF cytology. On the contrary approximately 22% of cases were cytology-negative and could only be detected by MRI and correlation with the clinical picture. Most probably the MRI-negative cases reflect diffuse fluid types while cytology-negative cases primarily represent adherent types of NM. Taken together, adding the complementary method enhanced the overall diagnostic accuracy by approximately 20%, making each method indispensible. The high sensitivity of MRI is in line with other recent studies, while other authors reported a considerably lower sensitivity of CSF cytology [21].

In hematological malignancies, CSF-cytology was clearly more sensitive in detecting NM than MRI. The rate of 100% sensitivity in cases of elevated cell counts most probably is overestimated because no cytology-negative cases could be identified in this subgroup in spite of extensive search of the databases. In any case, the difference to MRI sensitivity is striking and in line with the findings of other groups. In a large series of primary CNS lymphoma, Fischer et al. found an even lower rate of 4–18% of cases identified by MRI [25, 26]. Similar results have been achieved by other groups [21]. This may be explained by the biological characteristics of cells of hematological cells origin which are not designated for adherence to and formation of tissues. Therefore, these cells will rather float freely in the CSF than adhere to the meninges. By contrast, malignant cells of epithelial origin are much more likely to adhere and form layers of neoplastic tissue with permeable vasculature that can be detected by MRI [27, 28].

**Figure 1:**
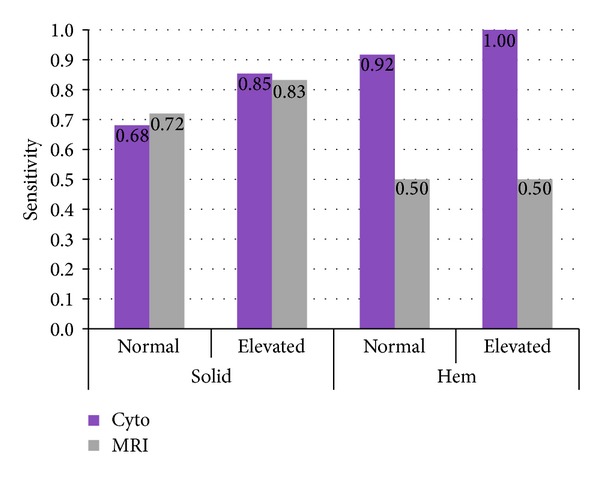
Diagnostic sensitivities of CSF cytology (“cyto”) and MRI, illustrated separately for solid (left) and elevated cell hematological (“hem”) (right) malignancies. and for normal and elevated cell counts.

In addition to the results of previous series, we demonstrate here that the diagnostic sensitivities are considerably higher when the CSF cell count is elevated (Figure 1). Since one-third of these cases of NM had normal cell counts, special emphasis has to be given to detect these cases where results may only be slightly abnormal. These may reflect the early stages of disease which are expected to be the most promising for successful treatment.

**Figure 2:**
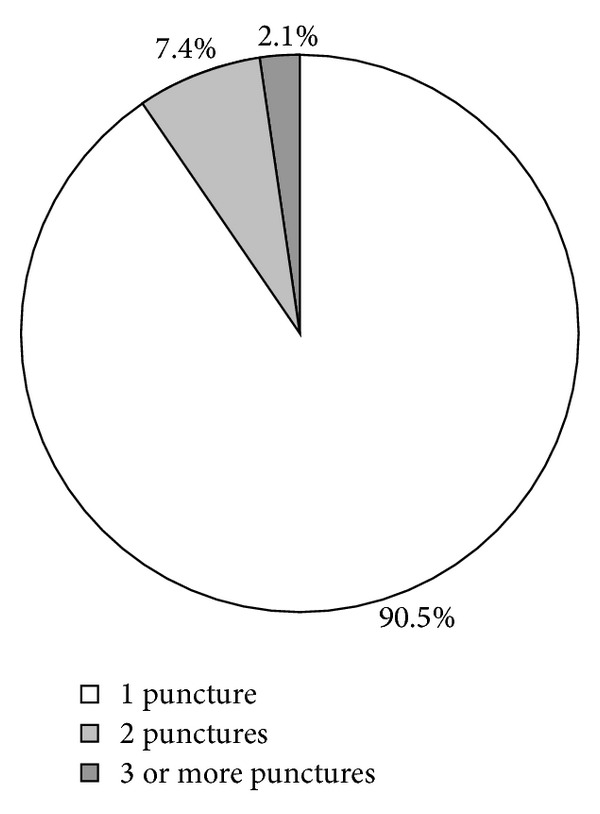
Numbers of CSF samples needed to establish the cytological diagnosis. Most of the diagnoses could be established already with the first puncture, while the second and consecutive samples only added a minor part of additional diagnoses.

Another scope of this study was to analyse how many CSF samples are needed to achieve an accuracy of CSF cytology of more than 90% as reported previously [29] (Figure 2). In this series, 91% of all cytology-positive cases were already diagnosed with the first and 98% with the second sample. All further lumbar punctures added only 2% additional positive cytologies. This translates into an overall sensitivity of 79% of all cases with the first puncture, 85% with the second and 87% with three or more samples. We therefore conclude that after 2 cytology-negative samples, there is only a minimal probability of a positive result in additional samples. Most interestingly, we found that the diagnostic accuracy was almost doubled from 55% to 94% since cytological diagnoses were established by two experienced cytologists in cooperation (IN and HS, starting from 2001) together with a close clinical follow-up of suspective cases.

As expected, the overall cell count and biochemical CSF analysis were unspecific and not suitable to detect NM. Surprisingly, one-third of the cases of NM reported here had normal CSF cell counts. This demonstrates that normal CSF cell counts cannot exclude malignant CSF disease. Interestingly, however, only 16% of all cases had completely normal standard CSF parameters, including total cell count, total protein, lactate, ferritin, and oligoclonal IgG.

We conclude that MRI is highly sensitive in detecting NM only in solid, but not in hematological malignancies. Since NM can occur with either cranial or spinal preference, high-resolution imaging of the whole crane and spine is mandatory to localize the spatial distribution of the disease. In addition, MRI is necessary for the detection of solid CNS metastases. CSF cytology can enhance the diagnostic accuracy by 12 to 50% depending on the diagnostic subgroup. In addition, repeated CSF analyses are suitable for the monitoring of treatment effects. Therefore, both methods are needed for the complete staging when NM is suspected.

## Figures and Tables

**Table 1:** Sensitivity for the detection of neoplastic meningitis.

Subgroup	MRI %	Cytology %	*P*
Whole group	71	87	<0.05
Normal cell count	59	76	>0.05
Elevated cell count	72	91	>0.05
Hematological	49	97*	<0.001
Solid	80	78	>0.05
Normal cell count hematological	50	92	>0.05
Elevated cell count hematological	50	100*	<0.001
Normal cell count solid	72	68	>0.05
Elevated cell count solid	83	85	>0.05

*The sensitivity of cytology in hematological neoplasms is artificially high for methodological reasons.

**Table 2:** Percentage of pathological values of standard CSF proteins in cases of neoplastic meningitis.

	All cases	Cell count		Neoplasm	
		Normal	Elevated	Solid	Hematol
Total protein	72	54	84	74	68
Lactate	52	59	46	19	73
IgG oligoclonal	42	36	44	30	42
Ferritin > 18 *µ*g/L	45	56	48	30	53
All normal	16				
